# Potential Therapeutic Targets of B7 Family in Colorectal Cancer

**DOI:** 10.3389/fimmu.2020.00681

**Published:** 2020-05-05

**Authors:** Changgang Wang, Haoran Feng, Xi Cheng, Kun Liu, Dongli Cai, Ren Zhao

**Affiliations:** ^1^Department of General Surgery, Ruijin Hospital North, Shanghai Jiao Tong University School of Medicine, Shanghai, China; ^2^Department of General Surgery, Ruijin Hospital, Shanghai Jiao Tong University School of Medicine, Shanghai, China; ^3^Clinical and Translational Research Center, Shanghai First Maternity and Infant Hospital, Tongji University School of Medicine, Shanghai, China

**Keywords:** colorectal cancer, immune checkpoint, B7-H3, VISTA, HHLA2

## Abstract

Programmed cell death protein 1 (PD-1)/programmed death ligand 1 (PD-L1) pathway blockade has impressively benefited cancer patients with a wide spectrum of tumors. However, its efficacy in colorectal cancer (CRC) is modest, and only a small subset of patients benefits from approved checkpoint inhibitors. Newer checkpoints that target additional immunomodulatory pathways are becoming necessary to activate durable antitumor immune responses in patients with CRC. In this review, we evaluated the mRNA expression of all 10 reported B7 family members in human CRC by retrieving and analyzing the TCGA database and reviewed the current understanding of the top three B7 family checkpoint molecules (B7-H3, VISTA, and HHLA2) with the highest mRNA expression, introducing them as putative therapeutic targets in CRC.

## Introduction

Colorectal cancer (CRC) is the fifth and the second most common cause of cancer mortality in China and the United States, respectively (1). The Global Cancer Statistics report estimated that over 1.8 million new CRC cases and 881,000 deaths occurred worldwide in 2018 (2). Although the outcomes of patients with CRC have improved in recent decades, mainly due to improvements in adjuvant/neoadjuvant therapy and surgical techniques, the survival rate remains poor, with a 5-year survival rate of 14% in patients with metastatic CRC (mCRC) (3). Novel agents and treatment regimens are thus urgently needed.

Similar to several other solid tumors, CRC is immunogenic (4), and immunotherapy, especially immune checkpoint blockade (ICB), has emerged as a promising strategy in the treatment of solid tumors (5), including CRC (6). In CRC, cytotoxic CD8^+^ tumor-infiltrating T cells (TILs) are the main effectors of antitumor immunity and function, considered one of the positive prognostic factors (7). However, the outcome of CD8^+^ T cell-based antitumor immunity is substantially controlled by immune checkpoints (including co-inhibitory and co-stimulatory molecules and their ligands) (8). By modulating the quantity and functional activity of antigen-specific T cells, immune checkpoint molecules play a pivotal role in mediating the critical bidirectional signals that control T cell activation and self-tolerance (9). In tumor progression, co-inhibitory checkpoint molecules are actively exploited to evade immunosurveillance (10), and a co-inhibitory immune checkpoint molecule blockade was confirmed to enhance the antitumor activity and maintain the highly durable antitumor immune responses of CD8^+^ TILs (11). Thus, artificial intervention strategies allowing these molecules to delay T cell exhaustion and improve the antitumor immune response in the tumor microenvironment (TME) have shown the clinical efficacy of cancer immunotherapy (6).

Given their proven value in numerous malignancies, programmed cell death protein 1 (PD-1) and its ligand PD-L1, the prototypical molecule in the B7 family, have been developed into eminent targets of antibody-based ICB therapies over the past few years (8). Despite the potent and durable antitumor effects found in a wide spectrum of tumors, only a minority of patients benefit from PD-1/PD-L1 pathway blockade (12). Indeed, the PD-1 inhibitors, nivolumab and pembrolizumab, have shown efficacy in a subset of mCRC patients with high levels of microsatellite instability (MSI-H) or mismatch repair deficiency (dMMR) (13). Since the rates of MSI-H or dMMR in CRC are only approximately 15% (11, 13), the vast majority of CRC patients with microsatellite stable (MSS) or mismatch repair proficiency or low levels of microsatellite instability (MSI-L) are ineligible for treatment with the current immune checkpoint inhibitors (13). This disappointing therapeutic efficacy indicates the urgent need for us to identify other checkpoint molecules of ICB in CRC.

**FIGURE 1:**
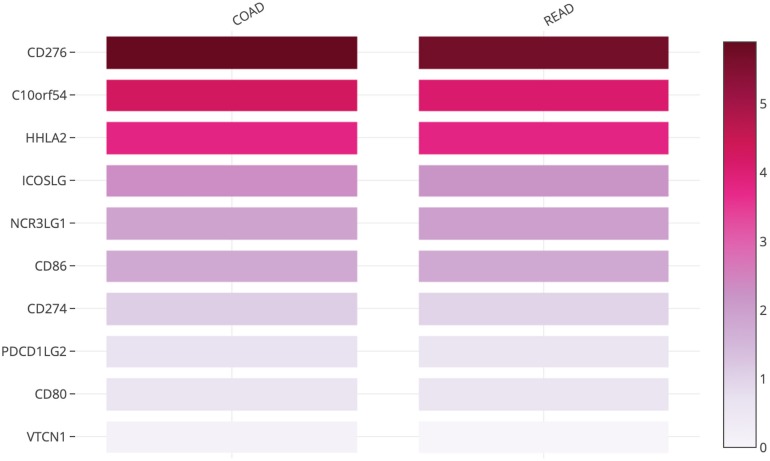
Heatmap analysis of the mRNA expression of B7 family members in CRC tumors shown as scaled log_2_-fold changes (GEPIA using data from TCGA).

**FIGURE 2:**
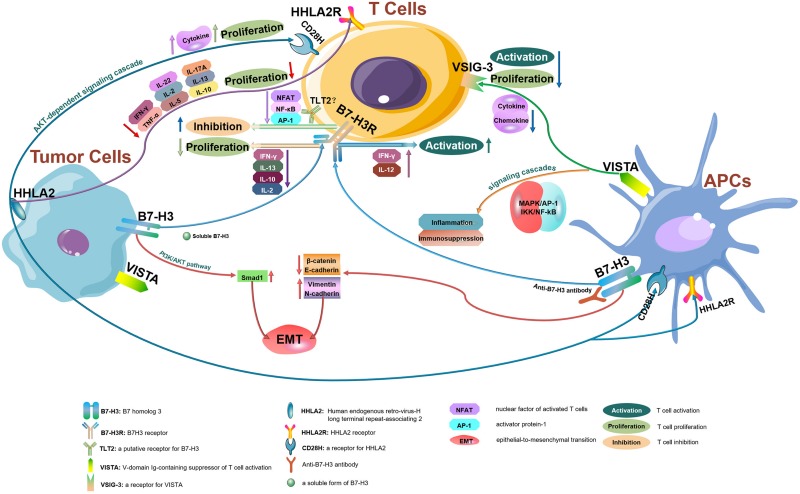
The regulatory mechanisms of B7-H3, VISTA, and HHLA2 in tumor microenvironments. B7-H3, VISTA, and HHLA2 are potential immune checkpoint molecules that interact with their receptors resulting in antitumor immune responses within tumor microenvironments.

The B7 family now comprises at least 10 reported members: CD80 (B7-1), CD86 (B7-2), PD-L1 (B7-H1), PD-L2 (B7-DC or CD273), ICOSL (B7-H2), CD276 (B7-H3), B7S1 (B7-H4, B7x or Vtcn1), VISTA (B7-H5, GI24, or PD-1H), B7-H6, and B7-H7 (HHLA2) (14). Using the TCGA database, we evaluated the messenger RNA (mRNA) expression levels of all B7 family members reported in human CRC. *CD276* (encoding B7 homolog 3, B7-H3), *C10orf54* (encoding V-domain Ig-containing suppressor of T cell activation, VISTA), and human endogenous retrovirus-H long terminal repeat-associating 2 (*HHLA2*, encoding B7-H7) (Figure 1) were found to be highly expressed in tumors from patients with CRC, indicating that these members may play important roles in CRC and account for the poor efficacy of the PD-1/PD-L1 ICB therapy for this cancer type. Therefore, in this review, we will focus on the recent advances in our understanding of B7-H3, VISTA, and HHLA2 in CRC; the regulatory mechanisms of these members are presented in the form of a pattern diagram (Figure 2).

## B7 Homolog 3

B7 homolog 3 (B7-H3, or CD276), a type I transmembrane glycoprotein encoded by chromosome 9 in mice and chromosome 15 in human, was identified as a homolog of the B7 family in 2001 which shares 20–27% amino acid identity with other B7 family members (15, 16). B7-H3 mRNA is ubiquitously expressed in most normal tissues, whereas its protein expression is relatively rare (14, 16). Overexpression of the B7-H3 protein is observed in a majority of human cancers, including CRC, and has been shown to be correlated with the poor outcomes of patients (17). Although several studies conclude that B7-H3 is involved in immunostimulatory regulation (15, 16), most studies describe B7-H3 as a co-inhibitory molecule (14, 18–21). The controversial roles of B7-H3 in immune regulation may be related to the unidentified B7-H3 receptor(s).

The B7-H3 protein has been found on the membrane and in the cytoplasm (22), within the nucleus and vasculature (23), as well as on interstitial infiltrating immune cells in CRC (24). A study including more than 700 CRC specimens showed that B7-H3 expression on the cytoplasm/membrane, stroma, or nucleus was seen in 86, 77, and 27% of the cases, respectively (23). In CRC patients, the varied intensity of B7-H3 was positively correlated with tumor grade (22) and negatively correlated with the intensity of TIL infiltration (22). Overexpression of B7-H3 was associated with the proliferation and invasion potential of tumor cells and was identified as a significant independent predictor of worse overall survival (OS) and disease-free survival (DFS) in patients with CRC (6). A study using tissue microarray analysis in CRC demonstrated that B7-H3 expression was correlated with clinical pathology and patient outcomes (23). Similarly, a meta-analysis including 1,202 total CRC cases showed that B7-H3 in CRC patients was obviously related to 24- and 72-month OS (25), demonstrating that CRC patients overexpressing B7-H3 may have a poor survival rate. In addition, nuclear localization of B7-H3 (23) and aberrant expression of B7-H3 in CD133^+^ CRC cells (26) were found to be closely associated with tumor progression and strongly predicted poor outcomes in CRC. B7-H3 also exists in a soluble form, which is cleaved from membranous B7-H3 by proteinase (15). Compared to that in healthy donors, the level of soluble B7-H3 in sera from CRC patients was upregulated, and TNF-α significantly increased the release of soluble B7-H3 in CRC cell lines (22), which indicates that soluble B7-H3 may be a potential prognostic marker for CRC. Therefore, both membranous and soluble B7-H3 are potentially associated with CRC progression and evasion of cancer immune surveillance.

B7 homolog 3 was suggested to serve as a conflicting co-inhibitory and co-stimulatory molecule in T cell regulation. Although the triggering receptor expressed on myeloid cell-like transcript 2 (TLT2) has been characterized as a putative receptor for B7-H3, additional or alternative receptors were suggested as indispensable for regulating the magnitude of T cell responses (27). Initial studies found that B7-H3 enhanced IFN-γ production selectively in the presence of T cell receptor (TCR) signaling and promoted cellular immunity (15, 28). In a CRC-bearing mouse model, mice treated with adenoviral B7-H3 showed suppressed tumor growth and reduced the occurrence of secondary metastasis, with significantly higher frequencies of IFN-γ-producing CD8^+^ T cells and higher IL-12 levels than those in the control group (29, 30). Similar results were also observed in an orthotopic CRC mouse model implanted with stable B7-H3-transfected colon-26 cell line (30). Accumulating evidence shows the idea that B7-H3 plays a negative rather than a positive regulatory role in antitumor immunity (18–21). By inhibiting or modulating nuclear factor of activated T cells (NFAT), nuclear factor-κB (NF-κB), and activator protein-1 (AP-1), B7-H3 may regulate TCR-mediated gene transcription and induce T cell inhibition (31). During T cell activation, B7-H3 showed potent and consistent inhibition of T cell proliferation and IFN-γ, IL-13, IL-10, and IL-2 production (19). *Via* a B7-H3-Ig fusion protein, B7-H3 was found to inhibit the proliferation of both CD4^+^ and CD8^+^ T cells in a dose-dependent manner. B7-H3 signal blockade augmented the responses of TH1 cells, but not TH2 cells or antiviral cytotoxic T lymphocytes (CTLs) (21). In a head and neck squamous cell carcinoma (HNSCC) mouse model, B7-H3 blockade significantly reduces myeloid-derived suppressor cells (MDSCs) and tumor-associated macrophages (TAMs), as well as promotes the IFN-γ secretion of cytotoxic T cells (32). The role of B7-H3 in regulating TILs may be dependent on the context of the TME, which is difficult to study because its binding partner(s) are unknown. In E.G7- and MOPC315-bearing mouse models, B7-H3 on antigen-presenting cells (APCs), but not on tumor cells, was claimed to account for the immunosuppression function. APC-expressed B7-H3 was reported to potently inhibit CD8^+^ T cell and natural killer (NK) cell activation. B7-H3-deficient mice or mice treated with an anti-B7-H3 antibody showed a significantly delayed tumor growth (18). However, an article published recently reported that in an ID8-bearing ovarian cancer model, *via* attenuating the expansion and cytotoxicity of CD8^+^ TILs, the tumor cell-expressed B7-H3 plays a predominant role in suppressing antitumor immunity. Host deletion of *Cd276* showed no significant difference in tumor growth compared with wild-type mice in an ID8-bearing mouse model (33). Similar phenomena were also found in MC38 colon, SW620 colon, and UACC melanoma-bearing mice (34). Furthermore, the synergic effects of the dual blockade of PD-1 with B7-H3 appear to be affected in the context of the TME, which results in addictive effects in an E.G7 model, but not in an ID8 model (33). In non-small-cell lung carcinomas (NSCLCs), B7-H3-negative tumors demonstrated abundant CD8^+^ TIL infiltration, and an anti-B7-H3 antibody combined with anti-PD-1 antibody therapy showed potent antitumor activation in a Pan02 murine NSCLC model (35). Additionally, upregulated B7-H3 expression has also been associated with suppressed NK cell-mediated cell lysis (18). In glioma, both soluble and membranous B7-H3 were able to exert a protective role on NK cell-mediated tumor cell lysis (36). Moreover, in CRC, B7-H3 expression was positively related to the density of TAMs. During TAM differentiation, B7-H3 promoted the polarization of type 2 macrophages (M2) and converted the M1 phenotype to the M2 phenotype *via* the putative receptor(s) on the macrophages and monocytes (37).

B7 homolog 3 is broadly overexpressed by multiple tumor types on both cancer cells and tumor-infiltrating blood vessels while it is not detectable in normal tissues, making it a potential target of B7-H3-directed therapeutic agents. The injection of anti-B7-H3 drug conjugates into various human CRC xenografts simultaneously ablated B7-H3-positive tumor cells and the tumor vasculature and improved long-term OS (34). In a preclinical study, MAEE-linked anti-B7-H3 antibody–drug conjugates (ADCs) displayed a dose-dependent antitumor activity against B7-H3^+^ tumor cells in HCT-116, KM12, and HT29 colon, OVCAR3 ovarian, and MDA-MB-231 breast tumor xenografts. And pyrrolobenzodiazepine (PBD)-conjugated B7-H3 ADCs killed both tumor cells and tumor epithelial cells, eradicating established tumors and metastases and improving long-term OS in lung, colon, and breast cancers (34).

Beyond immune regulation, B7-H3 also has a crucial role in promoting epithelial-to-mesenchymal transition (EMT), invasion (38), metastasis (29), and chemotherapy resistance in CRC. Evidence has shown that B7-H3 upregulated Smad1 expression *via* the PI3K-Akt pathway (14), downregulated the expression of β-catenin and E-cadherin, and increased the expression of vimentin and N-cadherin, indicating that B7-H3 promotes EMT in CRC (39). By upregulating the Jak2–Stat3 signaling pathway, overexpression of B7-H3 not only elevated MMP-9, thus bestowing tumor cells with pro-migratory and pro-invasive abilities (40), but also reportedly contributed to apoptosis resistance in CRC cell lines (41). In addition, CRC cell-overexpressed B7-H3 upregulated the expression of X-ray repair cross-complementing group 1 (XRCC1) *via* the PI3K-AKT pathway and BRCA1/BRCA2-containing complex subunit 3 (BRCC3), which then repaired oxaliplatin (L-OHP) or 5-fluorouracil (5-FU)-induced DNA damage (42, 43). Furthermore, B7-H3 has been implicated in aberrant metabolic reprogramming (24). Through sterol regulatory element-binding protein (SREBP), B7-H3 has been reported to regulate the fatty acid synthase (FASN) gene, thus exerting effects on lipids in lung cancer. The co-expression of isocitrate dehydrogenase 1 (IDH1), which functions as a transcriptional target of SREBP, and B7-H3 is significantly correlated with the prognosis of CRC patients and may serve as a predictive marker (24). By promoting hexokinase 2 (HK2) expression in CRC cells, upregulated B7-H3 effectively increased aerobic glycolysis and promoted chemoresistance. Knockdown of B7-H3 or blockade of HK2 reversed the induced increase in glucose metabolism and endowed chemoresistance (44).

## V-Domain Ig-Containing Suppressor of T Cell Activation

V-domain immunoglobulin (Ig)-containing suppressor of T cell activation (VISTA), also known as PD-1 homolog (PD-1H), DD1α, C10orf54, B7-H5, GI24, Dies1, and SISP1, is a type I membrane protein consisting of a single N-terminal Ig V domain and an extracellular domain homologous to PD-L1 that was identified in 2011 (45). VISTA is most conserved among the B7 members and shows 90% identity between mice and human (46). Similar to mouse VISTA, human VISTA is predominantly, if not exclusively, expressed in the hematopoietic compartment, with the highest expression on myeloid cells (including patrolling and inflammatory monocytes), and lymphoid and myeloid dendritic cells (DCs), and reduced expression on CD4^+^ and CD8^+^ T cells and Tregs (47). VISTA expression has also been detected on tumor-infiltrating lymphocytes (48). In CRC, VISTA was confirmed to be expressed at high levels in tumor sections than in paracancerous tissues and normal tissues (1), and the high expression of VISTA was associated with poor survival (49). In the CRC TME, VISTA was predominantly expressed on tumor-infiltrating lymphocytes, mainly on MDSCs and monocytes (1). Moreover, VISTA has also been detected on pan-cytokeratin^+^ CD45^–^ CRC cells, although the CRC cell line SW620 showed no VISTA expression (1). VISTA mRNA expression in CRC was significantly correlated with the genes responsible for tumor immune invasion (1).

V-domain immunoglobulin (Ig)-containing suppressor of T cell activation has been recognized as a co-inhibitory immune checkpoint molecule that suppresses T cell activation directly *in vitro* and *in vivo* (50). A soluble VISTA-Ig protein or APCs expressing full-length VISTA acted as ligands to inhibit T cell proliferation and cytokine production *in vitro*, which could be inhibited by a VISTA-specific monoclonal antibody (51). In a mouse model, VISTA-overexpressing tumor cells suppress antitumor immunity, and VISTA blockade exacerbates the development of T cell-mediated autoimmune encephalomyelitis (51). VISTA reportedly functions as a co-inhibitory receptor for CD4^+^ T cells, and a VISTA-specific agonistic mAb directly inhibits CD4^+^ T cell activation (52). In renal cell carcinoma (RCC), VISTA has been found highly expressed on intratumoral myeloid cells and strongly correlated with poor CD8^+^ T cell response. Blockade of VISTA suppressed the tumor growth significantly in a murine RENCA RCC model (53). Recently, VISTA was reported to inhibit human T cell proliferation as well as cytokine and chemokine production *via* VISG-3/VISTA signals, as VISG-3/IGSF11 was reported to be a ligand of VISTA expressed on activated T cells (48). VSIG-3 expression is upregulated in CRC and intestinal-type gastric cancers. Blockade of VSIG-3 by small interfering RNA (siRNA) blunted the growth of gastric cancer cells (48), suggesting that VSIG-3/VISTA may be a good immunotherapeutic target for gastrointestinal tumors with robust VISG-3 expression. In addition, VISTA has been found to control myeloid cell-mediated inflammation and immunosuppression by reducing the Toll-like receptor (TLR)-mediated activation of the MAPK/AP-1 and IKK/NF-κB signaling cascades. Blocking VISTA augmented pro-inflammatory mediator production and diminished the T cell-suppressive functions of myeloid cells, which resulted in a stimulatory TME and promoted T cell infiltration and activation (54). Moreover, in the CT26 murine CRC model, *via* HIF1α binding to the VISTA promotor, hypoxia upregulated VISTA expression preferentially on MDSCs, which in turn contributed to MDSC-mediated T cell suppression under hypoxic conditions (49).

Although the underlying mechanism is unclear, some links appear to exist between the expression patterns and functions of PD-1 and VISTA. In melanoma patients, PD-1/PD-L1 inhibitor treatment results in the upregulation of VISTA, which leads to adaptive resistance to the PD-1 blockade (1). Spontaneous T cell activation and chronic inflammation have been reported in VISTA or PD-1 single-knockout mice, whereas this phenotype is enhanced in PD-1/VISTA double-knockout mice (47). The magnitude of the T cell responses after challenge with foreign antigens is synergistically increased in PD-1/VISTA double-knockout mice compared with that in single-knockout animals (47). Furthermore, in the CT26 CRC mouse model, targeting VISTA and PD-L1 simultaneously rather than individually achieves a better tumor-clearing therapeutic efficacy (47).

## Human Endogenous Retrovirus-H Long Terminal Repeat-Associating 2

Human endogenous retrovirus-H long terminal repeat-associating 2 (HHLA2) is a newly identified member of the B7 family that shares 10–18% of its amino acids and 23–33% similarity to other human B7 proteins (55). HHLA2 is a type I transmembrane molecule with three extracellular Ig domains (IgV–IgC–IgV), which is unique because most other B7 family members have only two Ig domains (one IgV domain and one IgC domain), while B7-H3 contains four Ig domains (a tandem copy of the IgV–IgC domain) (56). HHLA2 was initially discovered as a gene in the Ig superfamily during a systematic search for human endogenous retrovirus (HERV) long terminal repeat (LTR) sequences that possessed enhancer, promoter, or polyadenylation functions (57). The LTR of the HHLA2 locus has been integrated into the primate lineage because it is found only in higher primates such as chimpanzees, gorillas, and humans and not in laboratory mouse or rat strains (31).

Human endogenous retrovirus-H long terminal repeat-associating 2 mRNA is broadly expressed in human normal tissues (58), while its protein level is restricted, except in the epithelia of the intestine, kidney, gallbladder, and breast and trophoblast cells of the placenta (59). The expression of HHLA2 in the intestines is considered to be important because it may control intestinal inflammation (59). In contrast to that in normal tissues, the protein expression of HHLA2 is widely observed in most of tumor specimens from the colon, breast, lung, thyroid, pancreas, ovary, liver, bladder, prostate, kidney, and esophagus, as well as in melanoma specimens (59). Several studies showed that overexpression of HHLA2 was significantly correlated with poor survival and clinical outcomes in patients with bladder urothelial carcinoma (60), clear cell renal cell carcinoma (61), osteosarcoma (62), and intrahepatic cholangiocarcinoma (63). However, high HHLA2 expression predicts better survival for patients with pancreatic cancer (64) and unresectable advanced or recurrent gastric cancer (65). A possible explanation for this discrepancy could be the difference in tumor type, tumor heterogeneity, clinical stage, or the methods used in the experiments. In CRC patients, the HHLA2 expression level was found to be positively correlated with a high mortality rate and significantly related to the depth of invasion and CD8^+^ T cell infiltration status and may thus act as an independent predictive factor associated with overall survival (66). Recently, HHLA2 was reported to be widely expressed in patients with PD-1-negative NSCLC, which suggests that HHLA2 might be a promising therapeutic target for patients who do not respond to PD-1 pathway blockade (67).

While HHLA2 is not inducible on T cells, it is constitutively expressed on human monocytes and macrophages and is induced on B cells after stimulation with lipopolysaccharide (LPS), IFN-γ, and poly I:C (55, 68, 69). HHLA2 is localized in both the cytoplasm and the membrane of tumor cells. As HHLA2 is a transmembrane protein, this type of distribution may be due to structural differences in staining or shuttling of the protein between the cytoplasm and the membrane (59). Currently, the detailed expression pattern of HHLA2 in CRC is still unclear.

In terms of the regulation of T cells, both co-stimulatory and co-inhibitory properties of HHLA2 have been described. When incubated with anti-CD3, an HHLA2 immunoglobulin fusion protein (HHLA2-Ig) what was reported to inhibit both CD4^+^ and CD8^+^ T cell proliferation and decrease the production of TNF-α, IFN-γ, IL-5, IL-10, IL-13, IL-17A, and IL-22 from T cells (55). HHLA2 also inhibits IL-2 secretion from T cells in a dose-dependent manner (31). Conversely, by binding to CD28H on T cells, HHLA2 has been reported to promote T cell proliferation and cytokine production *via* an AKT-dependent signaling cascade (68). In addition, in co-culture experiments, T cells from different donors also showed a heterogeneous functional response to HHLA2 protein in regard to cytokine production (55). The dual function of HHLA2 may depend on the immune milieu, receptor engagement/blockade, or interaction with different receptors.

Human endogenous retrovirus-H long terminal repeat-associating 2 can bind to its putative receptor(s) on T cells (resting and activated) and other immune cells, including B cells and APCs (31, 69), demonstrating that there are constitutive receptors on the cell surface. Transmembrane and immunoglobulin domain-containing 2 (TMIGD2, also known as IGPR-1 or CD28H) was identified as a receptor for HHLA2 (59). Similar to HHLA2, TMIGD2 was found in higher primates, but absent in laboratory mice and rats (70). TMIGD2 is widely expressed on DCs, monocytes, B cells, and naive T cells. Importantly, TMIGD2 was reportedly expressed only on naive CD4^+^ and CD8^+^ T cells and has been shown to rapidly disappear when the naive T cells are activated and begin the maturation phase (64). As tumor-infiltrating immune cells are activated cells, the crosstalk between TMIGD2 and HHLA2 is unlikely to explain the inhibition of the antitumor immune response, and new receptor(s) for HHLA2 need to be identified in the future.

Thus far, research about HHLA2 is still very rare, and research about HHLA2 in CRC is lacking, which may be related to the lack of expression of HHLA2 in laboratory mice. Because of its high expression, both at the mRNA and protein levels, in CRC, HHLA2 may be a potential immunotherapeutic target for CRC patients.

## Conclusion

In summary, ICB offers a novel strategy to eliminate or suppress tumor cells by generating an antitumor immune response. The clinical value of PD-1/PD-L1 inhibitors has already been demonstrated in some patients with cancer. However, in a majority of CRC patients, especially the patients who are microsatellite stable (MSS) or mismatch repair proficient or have low levels of microsatellite instability (MSI-L), the current immune checkpoint inhibitors appear to be ineffective. Additional insight into potential strategies for the vast majority of CRC patients is needed. At the mRNA level, tumors from CRC patients highly express B7-H3, VISTA, and HHLA2. Based on their robust expression and functional roles in cancer, these members appear to be promising targets for immune-based CRC therapies. Although significant progress has been made in the understanding of B7-H3, VISTA, and HHLA2, many important questions remain in CRC. The challenges with these members have been known to be receptor identification and the lack of expression of HHLA2 in laboratory mice. In addition, CRC is a complex and unique disease that contains the highest density of microorganisms in the human body. Reproducible changes in gut microbial diversity are observed during CRC progression and are associated with specific pathological features of tumors (38, 71). Thus far, research about the interaction of gut microbes with immune checkpoints is still lacking. Therefore, additional studies are needed, especially in primary human tumor specimens, and additional immunoregulatory mechanisms are needed. Hopefully, this increase in knowledge will lead to improved immunotherapeutic strategies to overcome the insensitivity of CRC to current immunotherapies.

## Author Contributions

CW and HF contributed to the conception of the article. XC, KL, DC, and RZ wrote and revised the final manuscript and agreed on its submission to this journal.

## Conflict of Interest

The authors declare that the research was conducted in the absence of any commercial or financial relationships that could be construed as a potential conflict of interest.
